# Monitoring and Regulating Intracellular GPX4 mRNA Using Gold Nanoflare Probes and Enhancing Erastin-Induced Ferroptosis

**DOI:** 10.3390/bios12121178

**Published:** 2022-12-17

**Authors:** Xiaoyan Liu, Qiangqiang Yang, Yanan Sui, Qiaoli Yue, Shuqing Yan, Chuan Li, Min Hong

**Affiliations:** 1 School of Chemistry and Chemical Engineering, Liaocheng University, Liaocheng 252059, China; 2 Shandong Harway Pharma Co., Ltd., Dongying 257000, China

**Keywords:** ferroptosis, GPX4 mRNA, AuNF probe

## Abstract

Glutathione peroxidase 4 (GPX4) plays an important effect on ferroptosis. Down-regulating the expression of GPX4 mRNA can decrease the content of GPX4. In this work, a gold nanoflare (AuNF) probe loaded with anti-sense sequences targeting for GPX4 mRNA was designed to monitor and down-regulate intracellular GPX4 mRNA using fluorescence imaging in situ and using anti-sense technology. The results revealed that there was a marked difference for the expression of GPX4 mRNA in different cell lines, and the survival rate of cancer cells was not significantly effected when the relative mRNA and protein expression levels of GPX4 was down-regulated by AuNF probes. However, when co-treated with AuNF probes, the low expression of GPX4 strengthened erastin-induced ferroptosis, and this synergy showed a better effect on inhibiting the proliferation of cancer cells.

## 1. Introduction

In the foreseeable future, cancer may replace cardiovascular disease as the disease that is causing the most deaths worldwide. As a result, researchers have conducted a lot of research work for the anti-cancer field. The present progress of anti-cancer drugs, treatment strategies, surgical technology, and imaging technology has significantly improved the survival rate of cancer patients, and many treatment methods aside from chemotherapy, radiotherapy, and surgery have gradually emerged, such as immunotherapy and gene therapy [1,2]. However, there is still no effective treatment to cure most cancers. Thus, it is quite urgent to develop new and effective cancer treatment methods.

With the development of modern technology, people have a deeper understanding of cell death. In addition to apoptosis, researchers have also found some other abnormal forms of cell death, such as autophagy, necrosis, pyroptosis, ferroptosis, etc. Among them, ferroptosis, a regulatory and iron-dependent programmed cell death form, has received extensive attention in recent years. Studies revealed that ferroptosis has some unique characteristics, such as shrinking mitochondria, increasing membrane density of mitochondria, and aggregation of iron and a series of destructive reactive oxygen species (ROS, including hydroxyl free radicals, lipid free radicals, and lipid hydrogen peroxide free radicals) [3,4]. ROS can oxidize intracellular nucleic acids, proteins, lipids, and other macromolecules. Especially, they will cause the oxidation of cell membrane lipids, which will eventually promote ferroptosis [3,5]. An effective ferroptosis inducer such as erastin can inhibit the cysteine/glutamate transporter system Xc-, leading to the depletion of cysteine (Cys) and glutathione (GSH) [6,7,8], further disrupting cellular redox homeostasis, inducing ROS accumulation, and then leading to the occurrence of ferroptosis.

In addition to inducing ferroptosis in cancer cells by elevating intracellular ROS levels, numerous studies have demonstrated the effectiveness of cancer-killing by inducing ferroptosis through the inactivation of glutathione peroxidase 4 (GPX4). GPX4 is a membrane lipid repair enzyme that can reduce lipid peroxide in the process of transforming GSH into GSSG, so it is a key factor to protect cells from ferroptosis [9,10]. Therefore, the inhibition of GPX4 is a key step in the process of exploring the role that contributes to the ferroptosis of cancer cells. Many inhibitors of GPX4 have been widely reported, such as RSL3 [11], ML162 [12], and FINO2 [13]. However, these inhibitors have some obvious disadvantages, such as poor selectivity and poor pharmacokinetic properties. Thus, there is an urgent need to find a method with good biocompatibility, easy metabolism, and effective inhibition of GPX4 activity. Protein synthesis is all translated from mRNA. If gene regulation methods such as anti-sense oligonucleotide technology are used to silence and down-regulate the expression of intracellular GPX4 mRNA, the activity of GPX4 can be indirectly suppressed. 

In light of the above description, in this study we designed an anti-sense oligonucleotide sequence that can silence GPX4 mRNA. The anti-sense sequence was loaded on gold nanoflare probes (AuNF probes), which was firstly developed by Mirkin’s group [14]. After the endocytosis of AuNF probes, the fluorescent molecule Cy5-modified anti-sense sequence will directly hybridize with GPX4 mRNA and further down-regulate the expression of GPX4. Moreover, the inhibition of cancer cell proliferation was studied through down-regulation of GPX4 alone with AuNF probes or through the synergistic effect of erastin and AuNF probes. Based on this probe, we sought to achieve the purpose of visually down-regulating and in situ monitoring GPX4 mRNA. Meanwhile, in order to overcome the disadvantages of erastin’s poor water solubility and avoiding its obvious toxic and side effects, we hope to explore a synergistic strategy that can more effectively induce ferroptosis in cancer cells under the effect of a low concentration of erastin.

## 2. Materials and Methods

### 2.1. Materials

Chloroauric acid (HAuCl_4_·4H_2_O), potassium dihydrogen phosphate (KH_2_PO_4_), disodium hydrogen phosphate (Na_2_HPO_4_), sodium citrate (C_6_H_5_Na_3_O_7_), sodium chloride (NaCl), and potassium chloride (KCl) were purchased from Shanghai Chemical Reagent Company (Shanghai, China). Phosphate buffer (PBS) was freshly prepared (pH 7.4, 136.7 mM of NaCl, 2.7 mM of KCl, 8.72 mM of Na_2_HPO_4_, 1.41 mM of KH_2_PO_4_). Magnesium chloride solution (1 M), RNase inhibitor (DEPC), 3-(4,5-dimethylthiazole-2)-2,5- diphenyltetrazolium bromide (MTT), and Tween-20 were purchased from Sigma-Aldrich (St. Louis, MO, USA). Other reagents are commercially available. The DNA sequences used (Table 1) were synthesized and purchased from Sangon Biotechnology Co., Ltd. (Shanghai, China). A glutathione peroxide detection kit (NADPH method), BCA protein concentration assay kit, immunostaining reagents (including fixed solution, washing solution, and blocking solution), GPX4 primary antibody (GPX4 rabbit polyclonal antibody), and GPX4 secondary antibody [Cy3 labeled Goat anti rabbit IgG (H + L)], were all purchased from Shanghai Beyotime Biotechnology Co., Ltd. (Shanghai, China).

### 2.2. Instrumentation

Fluorescence spectra and intensities were obtained on an F-7000 spectrophotometer (Hitachi, Tokyo, Japan). Absorption spectral measurements were carried out on a UV-750 ultraviolet spectrophotometer (Perkin-Elmer, Waltham, MA, USA). Transmission electron microscope (TEM) images were taken using a JEM 2100 electron microscope (JEOL Ltd., Tokyo, Japan). The TEM was operated at an acceleration voltage of 200 kV, and a micro grid was used for sample suspension. Confocal laser scanning microscopic analysis (CLSM) was carried out on a ZEISS LSM 880 microscope. Reverse transcription fluorescence quantitative PCR (qTR-PCR) was performed on the Quantstudio 5 Applied Biosystems instrument (Waltham, MA, USA). An MTT assay was performed on a ELX808 microplate reader (BioTek, Winooski, VT, USA).

### 2.3. Experimental Sections

#### 2.3.1. Preparation of Gold Nanoparticles and Gold Nanoflare Probes

Synthesis of gold nanoparticles (AuNPs) was conducted according to the literature [15]. We heated 50 mL of HAuCl_4_ (1 mM) solution to 100 °C, and after full boiling, 10 mL of trisodium citrate (38.8 mM) solution was rapidly added. After that, the color of the solution will gradually turn dark red. After stirring for 15 min, the reaction was stopped, and the mixture was naturally dropped to room temperature and was stored at 4 °C for standby. 

Gold nanoflare probes (AuNF probes) were synthesized according to the following steps. Add 100 μL of DEPC water to HS-DNA and Flare-DNA (or Flare-DNA-N) at 1 OD, respectively, and vortex to mix thoroughly. Then, add 1.5 μL (100 mM) of TCEP to the SH-DNA to activate the sulfhydryl groups, and let it stand for 1 h at room temperature in the dark. Mix HS-DNA and Flare-DNA (or Flare-DNA-N) at a ratio of 1:1.2 to form a nucleic acid hybridization solution. Then, the mixture was heated at 90 °C for 5 min and further cooled slowly. After the formation of the HS-DNA/Flare-DNA (or Flare-DNA-N) duplex, the solution was divided into 2 equal parts, and 1.2 mL of AuNPs solution was added to each part. After mixing at room temperature for 24 h in the dark, 10 µL of Tween diluent was firstly added, followed by adding 33 µL of PBS buffer solution containing 2 M of NaCl. Next, the passivation reaction was carried out by adding 33 µL of PBS buffer solution containing 2 M of NaCl three times every 2 h, and we continued to mix for 48 h. All operations were performed under dark conditions. Then, we centrifuged (13,500 rpm/30 min) the solution to remove the free DNA and continue to wash twice with PBS buffer solution. Finally, 1 mL of PBS buffer solution was used to dissolve the precipitate to form the AuNF probe solution, which was stored at 4 °C and should be used up in one month. The concentration of AuNF probe solution was calculated by the Lambert–Beer law (*ε* = 2.7 × 10^8^ L·mol^−1^·cm^−1^).

Gold nanoflare probe analogs (AuNF probe analogs) were also prepared using the same process mentioned above. However, the sequences of DNA duplexes (HS-DNA’/Flare-DNA’) that was bound with AuNPs was different from those of HS-DNA/Flare-DNA but with the same amount of bases.

#### 2.3.2. Incubation of AuNF Probes with Target-DNA or Cell Lysis Solution

AuNF probes (1.5 nM) and Target-DNA of different concentrations (0, 2.5, 5, 10, 50, 100, 200, and 300 nM) were mixed in PBS buffer solution to obtain 200 μL of solution, which was incubated at 37 °C for 4 h in the dark; we then measured the fluorescence intensity of each sample. 

The cell lysis solution was prepared by using an RNA extraction kit to extract RNA from 2 × 10^6^ HeLa cells and dissolve RNA in 100 μL of DEPC water. Then, take 20 μL cell lysis solution to mix with AuNF probes (1.5 nM) and produce up to 200 μL with DEPC water. Our mixture was incubated at 37 °C for 4 h in the dark and we then measured the fluorescence intensity.

#### 2.3.3. Cell Culture, Confocal Imaging, and Flow Cytometric Analysis

The medium (DMEM, GIBCO), fetal bovine serum, and double antibody [penicillin (100 μg·mL^−1^) and streptomycin (100 μg·mL^−1^)] were prepared according to the ratio of 9:1:0.1 to prepare the cell culture medium. The cells were cultured in a sterile environment at 37 °C humidified with 5% CO_2_.

Confocal laser scanning microscopy (CLSM) was used to detect the content of intracellular GPX4 mRNA through in situ fluorescence imaging after incubating AuNF probes (1.5 nM) for 4 h in 6 different cancer cell lines, including the human cervical cancer cell line (HeLa), human lung cancer cell line (A549), human hepatoellular carcinomas (HepG2), human breast cancer cell lines (MCF-7 and MDA-MB-231), human osteosarcoma cell line (U2OS); two normal cell lines were also used, including human normal liver cell line (HL-7702) and human normal breast cell line (HBL-100). We thoroughly mixed 200 μL of cell suspension (1 × 10^6^ mL^−1^) and 2 mL of cell culture medium and then inoculated them in a confocal dish. After culturing for 24 h, the AuNF probes (1.5 nM) were added. After incubating the cell samples in the incubator for 4 h, CLSM fluorescence imaging was determined using a 633 nm excitation. Then, the medium was removed, and the cells were trypsinized, collected, and washed twice with pre-chilled PBS. Apoptotic cells were detected by a Guava easyCyte flow cytometer. For each experiment, 5000 cells were recorded per sample.

#### 2.3.4. qRT-PCR Analysis

HeLa cells were seeded in 6-well plates, and three groups were set as the PBS group, control group (AuNF probe analogs), and AuNF probe group, which were prepared with Flare-DNA-N. The probes were added at different time points. After reaching the treatment time (12, 24, 36, 48 h), the culture medium was removed and thoroughly washed with PBS. Then, the cell samples were trypsinized and counted for extracting RNA according to the instruction of RNA extraction kit. After reverse transcription, they were stored in a −80 °C refrigerator for qRT-PCR detection. The contents of GPX4 mRNA were determined by qRT-PCR analysis using the SYBR Green Master Mix method. All primers used here are shown in Table 1.

#### 2.3.5. Immunofluorescence Labeling

HeLa cells were seeded in laser confocal dishes for 24 h; AuNF probes or PBS were added for 48 h. Then, the control and the AuNF probe groups were individually fixed, blocked, and incubated with primary antibody and Cy3-labeled secondary antibody according to the instruction of the assay kit. Finally, two groups were washed and observed under a confocal laser scanning microscope. To avoid the disturbance of Cy5 labeled on the Flare-DNA, AuNF probes used here were prepared with Flare-DNA-N. 

#### 2.3.6. Determining the Content of Glutathione Peroxidases

HeLa cells were seeded in cell culture dishes for 24 h and then treated with AuNF probes at different concentrations (0, 0.5, 0.8, 1.2, and 1.5 nM) for 48 h. With the same method, AuNF probes (1.5 nM) were added into HeLa cell samples and incubated for 0, 12, 24, 36, or 48 h. After washing the cells with PBS, the cells were digested on a small dish with 0.02% EDTA, then lyzed with a glass homogenizer. The protein concentrations of the cell lysis were determined with a BCA protein concentration assay kit and then detected according to the instruction of the glutathione peroxidase assay kit.

#### 2.3.7. Inhibiting the Proliferation of Cancer Cells through Erastin or the Synergistic Effect of Erastin and AuNF Probes

Five human cancer cell lines, including HeLa, A549, HepG2, MCF-7, and MDA-MB-231, were individually inoculated in 96-well plates (1 × 10^5^ cells/well). After incubating for 24 h, different volumes of erastin dissolved in DMSO were added into cell samples with the final erastin concentrations of 0.1, 0.5, 1, 2, 5, 10, and 15 µM. The cell viability of each sample was determined using the MTT method after interacting with erastin for 48 h. 

Similarly, five human cancer cell lines mentioned above were re-prepared in 96-well plates (1 × 10^5^ cells/well). Each cell sample was prepared in triplicate. A different cell sample was treated with AuNF probes (1.5 nM), erastin dissolved in DMSO (2 µM), or AuNF probes (1.5 nM) + erastin (2 µM), respectively. The cell viabilities of the cell samples were determined using the MTT method after being treated with different systems for 12, 24, or 48 h. 

#### 2.3.8. Determining the Content Change of Intracellular ROS Caused by Erastin and AuNF Probes

Three HeLa cell samples cultured in confocal dishes were individually prepared by adding HeLa cell suspension (200 μL, 1 × 10^6^ mL^−1^) and 2 mL of cell culture medium. After culturing for 24 h, AuNF probes were added into two cell samples with the final concentration of 1.5 nM. After 6 h, the medium containing AuNF probes was removed, and 2 mL of new cell culture medium with or without erastin (2 µM) were added. These two cell samples were set as the “AuNF probes” or “AuNF probes + erastin” group, respectively. After culturing for 48 h, two cell samples were stained with 2′,7′-dichlorodihydrofluorescein diacetate (DCFH-DA, 50 mM) in the medium for 30 min and then washed for three times with PBS solution and observed under a CLSM. The DCF was excited with a 488 nm laser and the fluorescence intensity at 525 nm shows the amount of DCF, which was correlated with the content of ROS.

The CLSM imaging of intracellular ROS for the only erastin-treated cell group was also dealt with in a similar process as the “AuNF probes + erastin” group, but without the treatment of AuNF probes.

#### 2.3.9. Statistical Analysis

All data were reported as the means ± standard deviation from at least three independent experiments. Statistical analyses were performed using the IBM SPSS Statistics 25 software package, and we assumed significance at *p* < 0.05 (*) and high significance at *p* < 0.01 (**).

## 3. Results and Discussion

### 3.1. Design of AuNF Probes and Mechanism of Enhancing Erastin-Induced Ferroptosis

**Figure 1:**
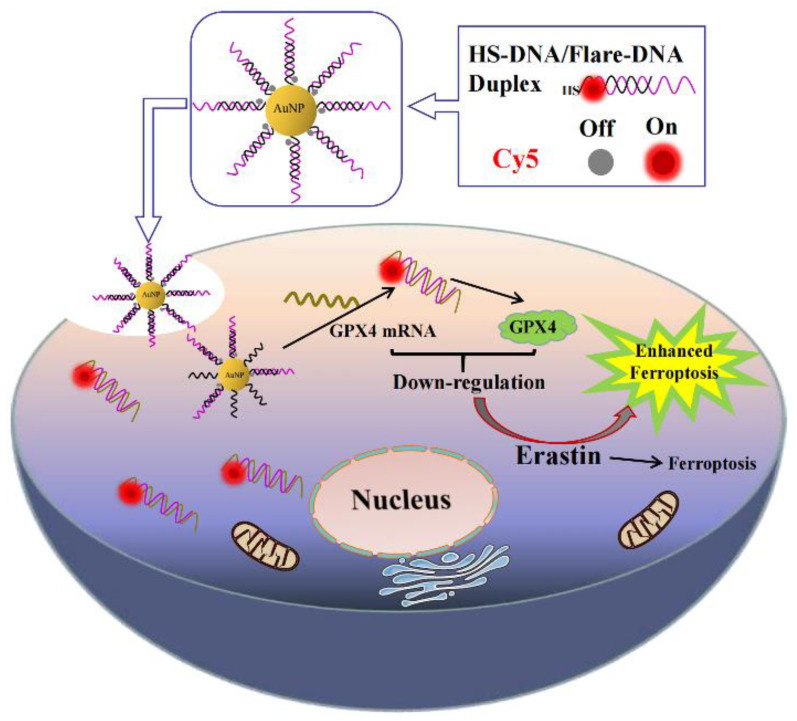
Schematic illustration of designation of AuNF probes, mechanism of down-regulating GPX4, and enhancement of erastin-induced ferroptosis for cancer cells.

Here, AuNF probes were prepared by the combination of HS-DNA/Flare-DNA duplexes and AuNPs through Au-S bonds. Flare-DNA was designed as the anti-sense sequences targeting for GPX4 mRNA and modified by the Cy5 fluorophore molecules at the 5′ end. Firstly, HS-DNA and Flare-DNA were co-incubated to form the HS-DNA/Flare-DNA duplexes, and the fluorescence signal of Cy5 was not effected. Once in the presence of AuNPs, AuNF probes were formed and the signal of Cy5 would be bleached due to the effect of the fluorescence resonance energy transfer (FRET). As shown in Figure 1, when the AuNF probes were endocytosed into the cell, intracellular GPX4 mRNA could competitively hybridize with Flare-DNA to form Flare-DNA/GPX4 mRNA hybridization systems, which will silence or degrade GPX4 mRNA and further down-regulate the expression of GPX4. At the same time, and the fluorescence signal of Cy5 is recovered due to the detachment of Flare-DNA from the surface of AuNPs. So, the above process of detecting and regulating GPX4 mRNA can be monitored by the fluorescence signal changes. In addition, the down-regulation of GPX4 was used to enhance erastin-induced ferroptosis for cancer cells.

### 3.2. Characterization of AuNPs and AuNF Probes

**Figure 2:**
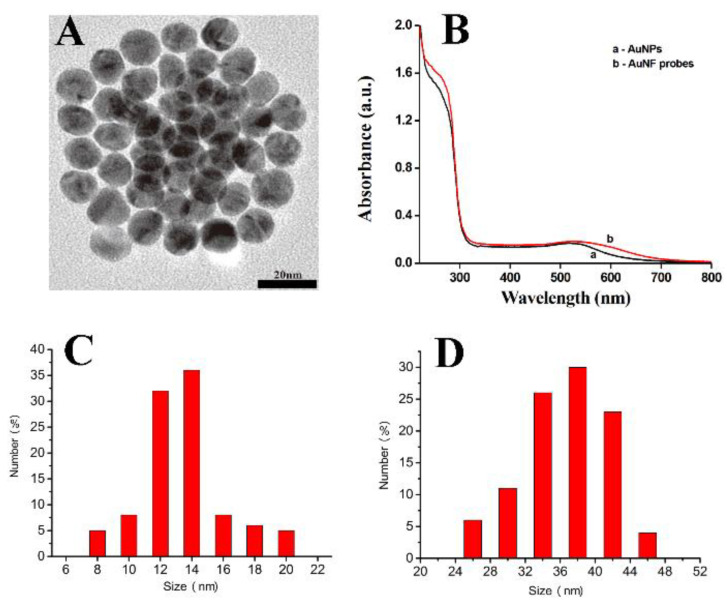
TEM images of AuNPs (**A**), UV-vis characterization of AuNPs and AuNF probes (**B**), hydrodynamic sizes of AuNPs (**C**) and AuNF probes (**D**) determined by DLS.

The size of AuNPs (~13 nm) was characterized by using TEM imaging (Figure 2A). The UV-vis spectra showed the characteristic absorption of AuNPs (~520 nm) and the DNA signal (~260 nm) for AuNF probes (Figure 2B). In addition, DLS was used to verify changes in the hydrodynamic size of AuNPs after binding with HS-DNA/Flare-DNA duplexes. As shown in Figure 2C,D, the hydrodynamic size of AuNF probes is 36 ± 0.3 nm, which is bigger than that of bare AuNPs (13.4 ± 0.4 nm). These results was basically consistent with relevant works [14,16].

### 3.3. Fluorescence Response of AuNF Probes to Target-DNA and Cell Lysis Solution

**Figure 3:**
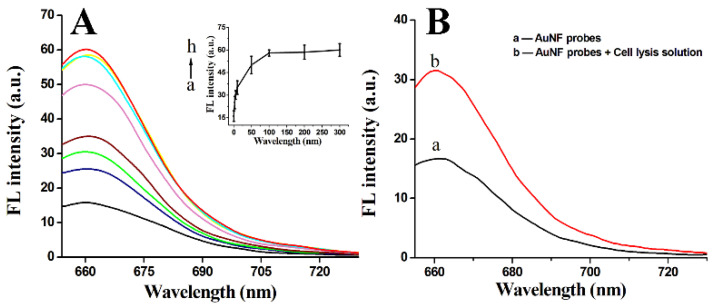
Fluorescence spectra of AuNF probes incubated with different concentrations of Target-DNA (0, 2.5, 5, 10, 50, 100, 200, and 300 nM) (**A**) or HeLa cell lysis solution (**B**).

According to the designation, the distance (-A_10_-, ~3.4 nm) of Cy5 and AuNPs in the AuNF probes is in the range of FRET [17]. So, there is weak fluorescence signals of Cy5 in the solution of AuNF probes (Figure 3). When the AuNF probes were treated with different concentrations of Target-DNA that were designed to completely hybridize with the Flare-DNA, the fluorescence signals of Cy5 were recovered. With the increase in Target-DNA concentrations, the fluorescence intensity increased accordingly, and there was a plateau when the concentration of the Target-DNA was above 100 nM (Figure 3A). These results show that the competitive hybridization between the Target-DNA and the Flare-DNA would induce a Flare-DNA detach from AuNF probes and recover the Cy5 fluorescence. A similar fluorescence recovery also occurred to the experimental system of AuNF probes and HeLa cell lysis solution (Figure 3B). Furthermore, to determine the specificity of the AuNF probes to the Target-DNA, Random-DNAs with arbitrary sequences were used to test the fluorescence response of AuNF probes. Studies show that the fluorescence intensity is quite small when AuNF probes incubate with high concentrations of Random-DNA (Figure S2). In addition, AuNF probe analogs have also been prepared by binding arbitrary sequence HS-DNA’/Flare-DNA’ hybridization duplexes on AuNPs. However, there was no obvious fluorescence response for the AuNF probe analogs to the Target-DNA (Figure S3A). All these results demonstrate the good specificity of the AuNF probes to GPX4 mRNA. 

### 3.4. Evaluation the Number of HS-DNA/Flare-DNA Duplexes Bound on AuNF Probes

According to the previous reports, the small molecule mercaptoethanol can compete with the nucleic acid hybridization duplexes and be bound to AuNPs by Au-S bonds. As AuNF probes (1.5 nM) were incubated with different concentrations of mercaptoethanol, and a sequentially enhanced fluorescence signal was observed with increasing mercaptoethanol concentration (Figure S1). This reveals that different amounts of HS-DNA/Flare-DNA duplexes were dissociated from the surface of the AuNPs. Based on the standard curve of HS-DNA/Flare-DNA duplexes, the amount of duplexes (~47) bound on each AuNF probe were evaluated by the fluorescence intensity of suspension solution of AuNF probes (1.5 nM) after being adequately treated with mercaptoethanol (50 μM) (Figure S1). Here, there are only 10 fully hybridized bases in the HS-DNA/Flare-DNA duplexes. Compared with previous similar gold nanoflare probes with 12 bases in the part of hybridization [16], the lower number of bound nucleic acid duplexes on the AuNF probe designed here should be due to the worse rigidity of the HS-DNA/Flare-DNA nucleic acid sequence caused by the lower number of hybridization bases.

### 3.5. Fluorescence Imaging of Intracellular GPX4 mRNA by CLSM

**Figure 4:**
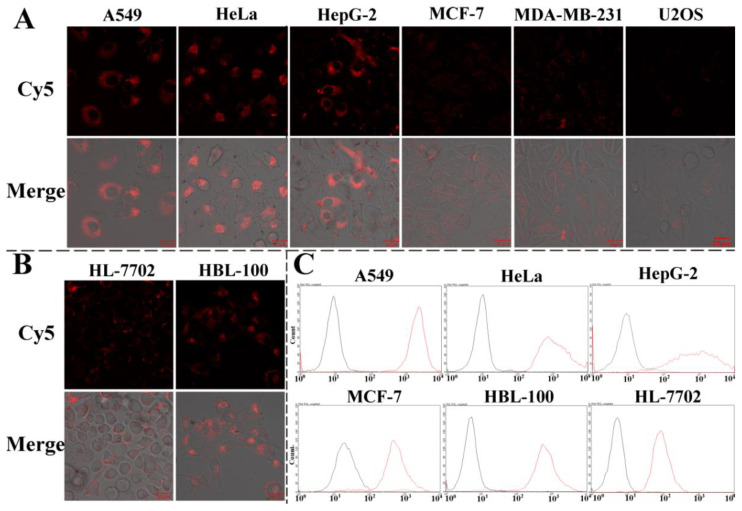
Fluorescence images of different cancer cell lines (**A**) and normal cell lines (**B**) that were treated with AuNF probes (1.5 nM) for 4 h. Flow cytometric analysis of different cell lines (**C**) collected after the CLSM determination.

Next, the response of the AuNF probes to intracellular GPX4 mRNA was examined by confocal laser scanning microscopy (CLSM). Six types of cancer cell lines were tested, including A549, HeLa, HepG2, MCF-7, MDA-MB-231, and U2OS. As shown in Figure 4A, there were great differences in the expression of GPX4 mRNA in the different cancer cell lines. A549, HeLa, and HepG-2 presented high expression. Both of the two types of breast cancer cell lines showed low expression. Similarly, there were slight fluorescence signals for U2OS, showing the low expression of GPX4 mRNA. At the same time, the expression of GPX4 mRNA in two normal cell lines were also determined. Compared with the HepG2 cells, the human normal liver cell line (HL-7702) presented a low expression of GPX4 mRNA, but normal breast cells (HBL-100) had higher levels of expression than their breast cancer cell counterparts (MCF-7 and MDA-MB-231) (Figure 4B). In addition, to further verify this phenomena, parts of the cell samples were collected for flow cytometric analysis. The general fluorescence signals were in accordance with the results determined by CLSM (Figure 4C). The content of GPX4 mRNA in HeLa, A549, MCF-7, and HBL-100 have also been tested by qRT-PCR. The data show similar results to those determined by the CLSM and flow cytometric analyses (Figure S4).

Moreover, the intracellular non-specific response of AuNF probes can be excluded by CLSM imaging for HeLa cells that were treated with AuNF probe analogs (Figure S3B,C). Compared with the stronger fluorescence intensity of the AuNF probe treatment group, the AuNF probe analog group only showed slight background signals.

### 3.6. Expression Analysis of Intracellular GPX4 mRNA, GPX4 and Total GPX

Recently, Hangauer et al. reported that drug-resistant persistent cancer cells are sensitive to GPX4. Ferroptosis of resistant cancer cells can be induced by inhibiting GPX4 with a direct GPX4 inhibitor (RSL3) [18]. In addition, Ni et al. used siRNA to down-regulate GPX4 mRNA to synergistically enhance ferroptosis of cancer cells when reacted with iron oxide and platinum nanocomposite systems [19].

**Figure 5:**
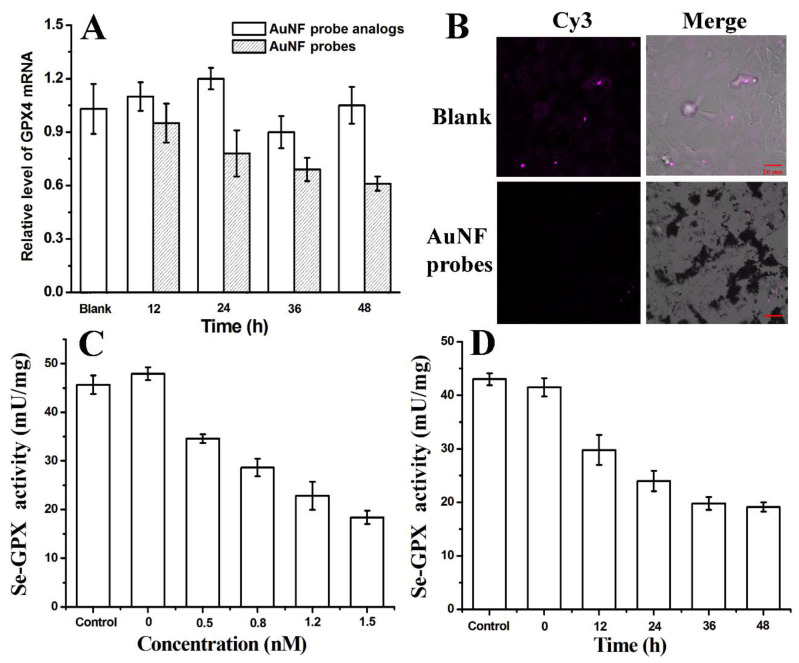
(**A**) qRT-PCR analysis of GPX4 mRNA in HeLa cells after being treated with AuNF probes (1.5 nM) or AuNF probe analogs (1.5 nM) for different times (12, 24, 36, and 48 h). (**B**) Immunofluorescence confocal imaging of GPX4 expression (purple) in HeLa cells after being treated with AuNF probes (1.5 nM) or PBS (Blank) for 48 h. (**C**) The activity of Se-GPX in HeLa cells after being treated with different concentrations of AuNF probes (0, 0.5, 0.8, 1.2, and 1.5 nM) for 48 h. (**D**) The activity of Se-GPX in HeLa cells after being treated with AuNF probes (1.5 nM) for different times (0, 12, 24, 36, and 48 h).

To further corroborate the critical role of GPX4, we down-regulated GPX4 mRNA expression in HeLa cells using anti-sense technology. The Flare-DNA sequences of AuNF probes were designed to completely hybridize with GPX4 mRNA. Once the AuNF probes enter the cells, Flare-DNA will hybridize with GPX4 mRNA to form a partial double-stranded structure. This hybridization will activate intracellular RNase H to hydrolyze GPX4 mRNA and reduce the content of intracellular GPX4 mRNA or to silence GPX4 mRNA to prevent its further translation into GPX4 protein [20]. To verify this effect, a qRT-PCR test was used to examine the content change of intracellular GPX4 mRNA when the AuNF probes reacted with HeLa cells for different times. With the increase in time, the GPX4 mRNA content showed a significant down-regulation trend, and it dropped to 61% at 48 h (Figure 5A). With a similar experimental operation, there was no obvious decrease for the expression of GPX4 mRNA in HeLa cells that were treated with the AuNF probe analogs. This result showed that AuNF probes could down-regulate the expression of intracellular GPX4 mRNA based on anti-sense technology.

The protein level of GPX4 was observed using the immunofluorescence labeling method with CLSM. According to the Cy3 fluorescence signals labeled on secondary antibody (Figure 5B), it can be seen that the GPX4 content in the AuNF probe group is significantly lower than that of the PBS group. These results indicate that AuNF probes can effectively down-regulate the expression of GPX4 protein after regulating intracellular GPX4 mRNA.

Furthermore, the activity of total GPX containing selenium (Se-GPX) were determined using a glutathione peroxidase assay kit with the NADPH method. As shown in Figure 5C,D, with the increase in AuNF probe concentration and the prolongation of reaction time, the activity of Se-GPX showed a downward trend, which was consistent with the results of the down-regulation of the expression of GPX4. 

### 3.7. Enhancing Erastin-Induced Ferroptosis Using the Synergistic Effect of AuNF Probes

Ferroptosis inducers can be divided into two types. The first type, such as erastin and sulfasalazine, acts by targeting cystinoglutamate anti-transporters (system Xc-). The second type, such as RSL3 and DP17, can directly inhibit GPX4 activity [21,22]. Both of these types of inducers can mediate intracellular ROS accumulation and inhibit the proliferation of cancer cells with the form of ferroptosis. 

**Figure 6:**
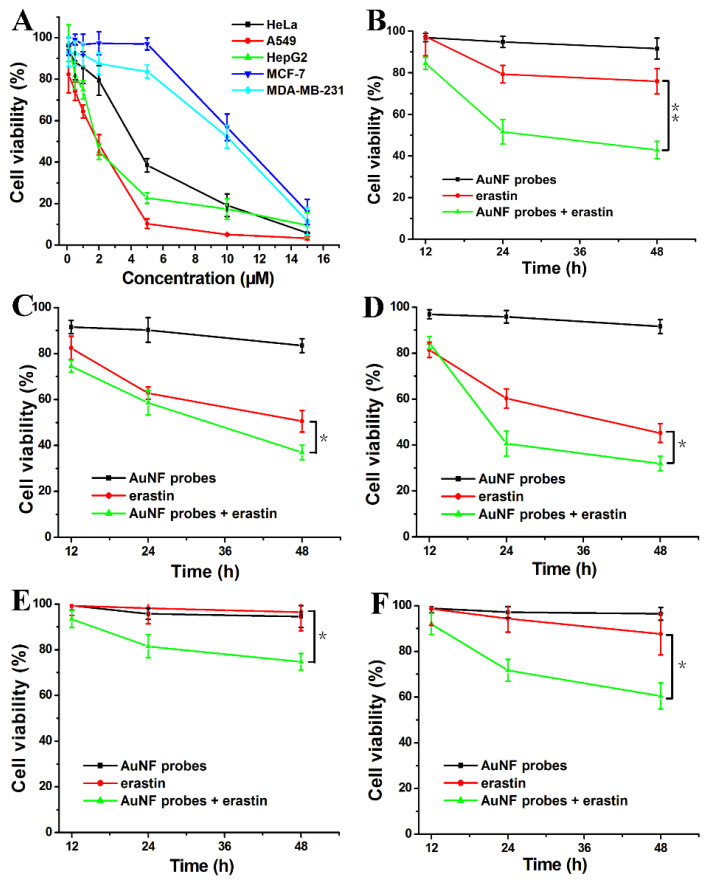
(**A**) Cell viabilities of different cancer cell lines when treated with different concentrations of erastin (0.1, 0.5, 1.0, 2.0, 5.0, 10.0, and 15.0 µM) for 48 h. Cell viability of HeLa (**B**), A549 (**C**), HepG2 (**D**), MCF-7 (**E**), and MDA-MB-231 cells (**F**) when treated with AuNF probes (1.5 nM), erastin (2.0 µM), and AuNF probes (1.5 nM) + erastin (2.0 µM) for different times (12, 24, and 48 h). *p* < 0.05 (*); *p* < 0.01 (**).

Here, we plan to study the inhibition of the proliferation of cancer cells while simultaneously using these two kinds of ferroptosis inducers. Erastin is a low molecular weight chemotherapy drug that can induce ferroptosis for different types of cancer cells [23,24,25]. Our experiments show that erastin exhibits obvious in vitro cytotoxicity against the five cancer cell lines, including HeLa, A549, HepG2, MCF-7, and MDA-MB-231 (Figure 6A). Especially for A549, the IC50 value is low, measuring at 2.32 µM. Overall, the inhibition rates of cell proliferation are dependent on the concentration of erastin. However, poor water solubility and renal toxicity have limited its clinical application. It was reported that the expression of GPX4 appeared to strongly correlate with the sensitivity to ferroptosis induction by erastin [12]. Compared with the above measured expression levels of GPX4 mRNA, we noticed that erastin showed significant cytotoxic activity at the lower concentration range (<5 µM) for HeLa, A549, and HepG2 cells, which presents a higher expression of GPX4 mRNA. The semi-inhibitory concentrations (IC50) are shown in Table S1. For MCF-7 and MDA-MB-231, both of their half-inhibitory concentrations were greater than 10 µM. All these results show the correlation of erastin-mediated ferroptosis with the expression of GPX4. 

Considering the low water solubility of erastin, the authors aimed to explore some new strategies to conjugate with other ferroptosis promoters to maximize the anti-cancer effect of erastin at low concentrations. The inactivation of GPX4 is believed to lead to increased levels of uncontrolled lipid peroxidation culminating in cell death in vitro and in vivo [12,18]. To further test the possibility and the correlation between the expression of GPX4 and erastin, we treated five cancer cell lines with AuNF probes for different times. We found that the AuNF probes show a weaker inhibition for the proliferation of the five cancer cell lines by only down-regulating GPX4 (Figure 6B–F). Of course, the cytotoxicity of AuNPs itself can be excluded by determining the cell viability of HeLa cells that were treated with different concentrations of AuNF probe analogs for 48 h (Figure S3D).

Compared with the cell viability of different cancer cells treated with erastin (2 µM) alone for different times, the co-treatment with AuNF probes and erastin has better inhibition rates for the proliferation of all cancer cell lines except for A549 cells under these unoptimized conditions. According to the inhibition rate of different systems on the proliferation of different cancer cells at 48 h (shown in Table S2), we can see that there exists a drug synergism of “1 + 1 > 2”. Thus, targeting system Xc- and directly inhibiting GPX4 activity can achieve a synergistic inhibition of cancer cell proliferation. 

### 3.8. ROS Generation Caused by AuNF Probes and Erastin

To further demonstrate the relationship of cytotoxicity of AuNF probes or erastin and their ability to generate ROS, the content change of intracellular ROS was evaluated by the CLSM analysis using DCFH-DA as the fluorescence molecular probe. 

**Figure 7:**
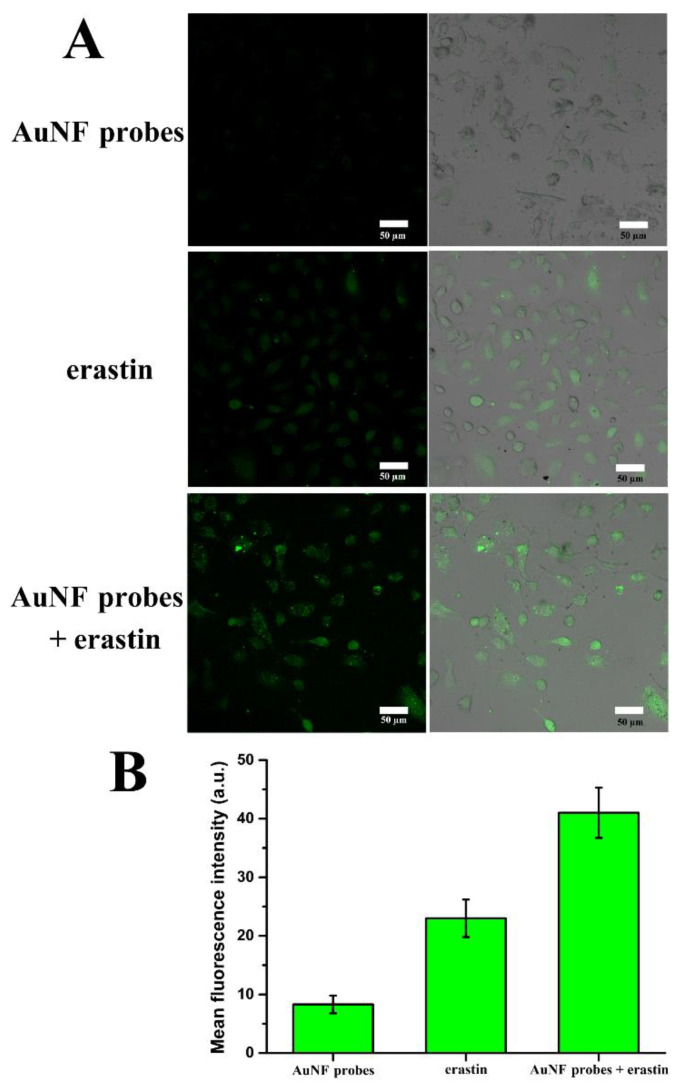
(**A**) Confocal images of HeLa cells showing the ROS response when cells were treated by different systems. (**B**) Mean fluorescence intensity of confocal images of HeLa cells shown in (**A**).

A large number of studies have shown that ROS is the key point of ferroptosis in cells [3,4,5], and GPX4 shows the ability to clear intracellular ROS [9,10]. Therefore, the down-regulation of GPX4 should lead to intracellular ROS accumulation, which induces cell death. Figure 7 shows the confocal images of HeLa cells when the cells were treated by the AuNF probes. Unexpectedly, there is only a slight fluorescence signal of DCA, which demonstrates a lower content of intracellular ROS. Compared with the inhibition of GPX4 activity shown in Figure 5, we speculated that the above results should be due to the poor inhibition rate (40% at 48 h) of GPX4 activity by the AuNF probes, so that the remaining GPX4 could still clear most of the intracellular ROS. This may also explain why AuNF probes had less effect on the survival of the cancer cells tested (Figure 6B–F).

For the erastin-treated cell sample, the confocal images show obvious fluorescence signals of DCA, which verify the previous report that erastin can mediate intracellular ROS accumulation [23,24,25]. Apparently, the stronger fluorescence signals for AuNF probes and erastin co-treated cell sample demonstrated that the synergistic effect of the AuNF probes and erastin more significantly promoted the accumulation of large amounts of ROS in HeLa cells. These results are in accordance with the greater cell inhibitory activity shown in Figure 6B–F.

## 4. Conclusions

At present, there are more and more studies on ferroptosis, which are inseparable from the study of GPX4. In this study, we constructed a AuNF probe for monitoring and down-regulating intracellular GPX4 mRNA based on anti-sense oligonucleotide technology. Studies reveal that the proliferation of cancer cells was not obviously effected only by down-regulating the expression of GPX4. Nevertheless, ferroptosis induced by erastin can be enhanced by the synergistic effect of the AuNF probe. In conclusion, this study provides a new research idea for promoting the clinical application of erastin and finding better anti-cancer strategies.

## Figures and Tables

**Table 1:** DNA sequence list.

Names	Sequences
HS-DNA	5′-TCA GCG TAT CAA AAA AAA AA-SH-3′
HS-DNA’	5′-GAT CTA AGC TAA AAA AAA AA-SH-3′
Flare-DNA	5′-GAT ACG CTG AGT GTG GTT T-Cy5-3′
Flare-DNA’	5′-AGC TTA GAT CGC TAT GTA C-Cy5-3′
Flare-DNA-N	5′-GAT ACG CTG AGT GTG GTT T-3′
Target-DNA	5′-AAA CCA CAC TCA GCG TAT C-3′
Random-DNA	5′-GGC CGA TTG TGA ACA TGG A -3′
GPX4-F-primer	5′-AGA GAT CAA AGA GTT CGC CGC-3′
GPX4-R-primer	5′-TCT TCA TCC ACT TCC ACA GCG-3′
GAPDH-F-primer	5′-CTC AGA CAC CAT GGG GAA GGT GA-3′
GAPDH-R-primer	5′-ATG ATC TTG AGG CTG TTG TCA TA-3′

## Data Availability

Not applicable.

## Supplementary Materials

The following supporting information can be downloaded at: https://www.mdpi.com/article/10.3390/bios12121178/s1, Figure S1: (A): Fluorescence recovery of AuNF-probes incubated with different concentrations of mercaptoethanol (0, 0.2, 0.5, 1, 20, 50 μM). (B): Standard curves of the fluorescence intensity of HS-DNA/Flare-DNA duplexes (0, 5, 10, 20, 40, 60, 80, 100, 150 nM). Figure S2: Fluorescence spectra of AuNF-probes incubated with different concentrations of Random-DNA (0, 100, 300 nM). Figure S3 (A): Fluorescence spectra of AuNF-probe analogs incubated with different concentrations of Target-DNA (0, 100, 300 nM). (B): Confocal fluorescence images of HeLa cells that were treated with or without AuNF-probe analogs (1.5 nM) for 4 h. (C): Mean fluorescence intensity of HeLa cells that shown in Figure S2B and Figure 4A. (D): Viabilities of HeLa cells when treated with different concentrations of AuNF-probe analogs (0.5, 1.0, 1.5, 2.0 nM) for 48 h. Figure S4: qRT-PCR analysis of GPX4 mRNA content in HeLa, A549, MCF-7, and HBL-100 cells. Table S1: IC50 values of erastin against five different cancer cell lines. Table S2: Inhibition rate of different systems on the proliferation of different cancer cells at 48 h.
