# Supplementary material for: Monitoring and Regulating Intracellular GPX4 mRNA Using Gold Nanoflare Probes and Enhancing Erastin-Induced Ferroptosis
Source: Biosensors (Basel). 2022 Dec 17;12(12):1178. doi: 10.3390/bios12121178 (PMC9776005; doi:10.3390/bios12121178)
Supplement: Supplementary file 1 [file biosensors-12-01178-s001.zip › biosensors-2042243-supplementary.pdf]

biosensors

Electronic Supporting Information

Monitoring and Regulating Intracellular GPX4 mRNA Using Gold Nanoflare Probes and Enhancing Erastin-Induced Ferroptosis

Xiaoyan Liu [1,†], Qiangqiang Yang [1,†], Yanan Sui [1], Qiaoli Yue [1], Shuqing Yan [1], Chuan Li [1] and Min Hong [1,2,*]

[1] School of Chemistry and Chemical Engineering, Liaocheng University, Liaocheng 252059, China
[2] Shandong Harway Pharma Co., Ltd., Dongying 257000, China
* Correspondence: hongmin@lcu.edu.cn; Tel.: +86-635-823-9195; Fax: +86-635-823-9121
† These authors contributed equally to this work.

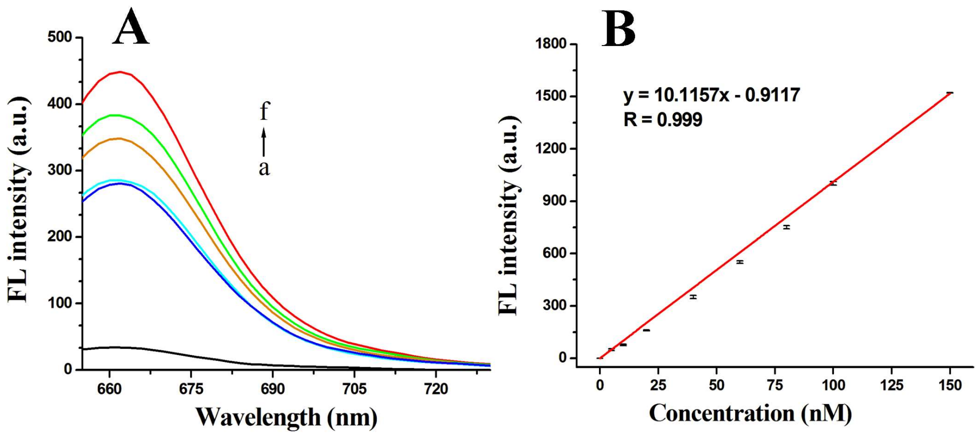

Figure S1. (A) Fluorescence recovery of AuNF-probes incubated with different concentrations of mercaptoethanol (0, 0.2, 0.5, 1, 20, 50 μM). (B) Standard curves of the fluorescence intensity of HS-DNA/Flare-DNA duplexes (0, 5, 10, 20, 40, 60, 80, 100, 150 nM).

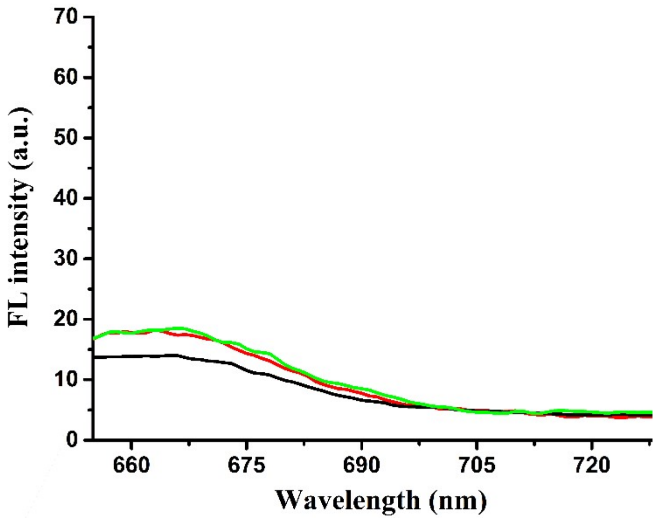

Figure S2. Fluorescence spectra of AuNF-probes incubated with different concentrations of Random-DNA (0, 100, 300 nM).

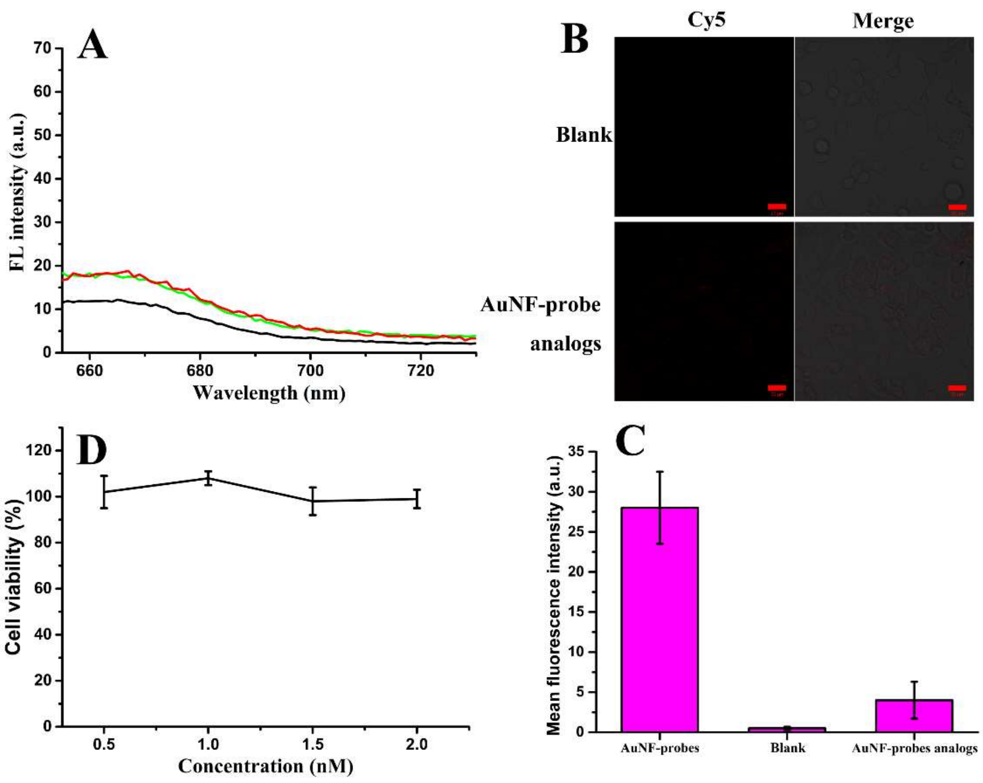

Figure S3. (A) Fluorescence spectra of AuNF-probe analogs incubated with different concentrations of Target-DNA (0, 100, 300 nM). (B) Confocal fluorescence images of HeLa cells that were treated with or without AuNF-probe analogs (1.5 nM) for 4 h. (C) Mean fluorescence intensity of HeLa cells that shown in Figures S2B and 4A. (D) Viabilities of HeLa cells when treated with different concentrations of AuNF-probe analogs (0.5, 1.0, 1.5, 2.0 nM) for 48 h.

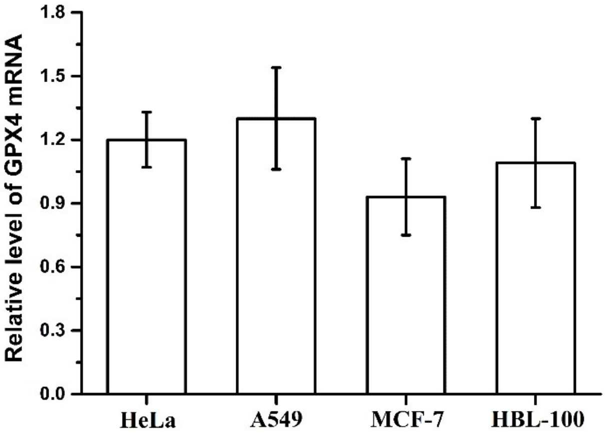

Figure S4. qRT-PCR analysis of GPX4 mRNA content in HeLa, A549, MCF-7, and HBL-100 cells.

Table S1. IC_{50} values of erastin against five different cancer cell lines.

	HeLa	A549	HepG2	MCF-7	MDA-MB-231
**erastin**	2.32 ± 0.34	5.41 ± 0.34	3.83 ± 0.64	10.89 ± 0.23	10.24 ± 0.42

Table S2. Inhibition rate of different systems on the proliferation of different cancer cells at 48 h.

	HeLa	A549	HepG2	MCF-7	MDA-MB-231
**AuNF-probes**	8.41 ± 5.06	16.62 ± 2.98	8.46 ± 3.06	5.58 ± 4.76	3.66 ± 2.76
**erastin**	24.12 ± 6.11	49.54 ± 4.72	54.81 ± 4.11	3.69 ± 8.15	12.58 ± 9.14
**AuNF-probes + erastin**	57.19 ± 4.17	63.18 ± 3.17	68.19 ± 3.17	25.32 ± 3.66	39.65 ± 5.69
